# Supplementary material for: Conformational coupling of redox-driven Na+-translocation in Vibrio cholerae NADH:quinone oxidoreductase
Source: Nat Struct Mol Biol. 2023 Sep 14;30(11):1686–94. doi: 10.1038/s41594-023-01099-0 (PMC10643135; doi:10.1038/s41594-023-01099-0)
Supplement: Supplementary file 1 — Supplementary Figs. 1–6 and Tables 1–4. [file 41594_2023_1099_MOESM1_ESM.pdf]

nature structural & molecular biology

Article https://doi.org/10.1038/s41594-023-01099-0

Conformational coupling of redox-driven Na^+-translocation in *Vibrio cholerae* NADH:quinone oxidoreductase

In the format provided by the authors and unedited

Supplementary Information

Conformational coupling of redox-driven Na^{+}-translocation in *Vibrio cholerae* NADH:quinone oxidoreductase

Jann-Louis Hau[1], Susann Kaltwasser[2], Valentin Muras[1], Marco S. Casutt[1], Georg Vohl[1], Björn Claußen[1], Wojtek Steffen[1], Alexander Leitner[3], Eckhard Bill[4], George E. Cutsail III[4], Serena DeBeer[4], Janet Vonck[5], Julia Steuber[1], Günter Fritz[1]

[1]Department of Cellular Microbiology, Institute of Biology, University of Hohenheim, 70599 Stuttgart, Germany

[2]Central Electron Microscopy Facility, Max Planck Institute of Biophysics, 60438 Frankfurt am Main, Germany

[3]Department of Biology, Institute of Molecular Systems Biology, ETH Zürich, 8093 Zürich, Switzerland

[4]Max Planck Institute for Chemical Energy Conversion, 45470 Mülheim an der Ruhr, Germany

[5]Department of Structural Biology, Max Planck Institute of Biophysics, 60438 Frankfurt am Main, Germany

Supplementary Information Table of Contents

Movement of ferredoxin-like domain of NqrF 3

Supplementary Figure 1. Structural snapshots of rotational flexibility of the ferredoxin-like domain of NqrF 4

Analysis of the intramembranous [2Fe-2S] cluster in NqrD-E 5

Supplementary Table 1. Detection of iron and acid-labile sulphide in wt Na^+-NQR and variants 5

Supplementary Table 2. Electron transfer activity of wt Na^+-NQR and variants 6

HERFD-XAS Fe K-edge and analysis of the [2Fe-2S] clusters in NqrF and NqrD-E 7

EPR spectroscopic analysis of the [2Fe-2S] clusters in NqrF and NqrD-E 7

Supplementary Figure 2 . HERFD-XAS Fe K-edge and EPR spectra of Na^+-NQR wildtype and Na^+-NQR variants 8

Supplementary Figure 2. HERFD-XAS Fe K-edge and EPR spectra of Na^+-NQR wildtype and Na^+-NQR variants 9

Supplementary Table 3. EXAFS Fit Parameters 10

Supplementary Figure 3. EXAFS of NqrF-C70A and NqrD-C29A 10

UV-visible absorption spectroscopy of wt-NQR and of a Na^+-NQR variant 11

Supplementary Figure 4. Spectroscopic and fast kinetic analysis of wt Na^+-NQR and Na^+-NQR variants reveal specific features of the $[2Fe\text{-}2S]_{NqrD\text{-}E}$ 11

The $[2Fe\text{-}2S]_{NqrD\text{-}E}$ centre is conducting transmembrane electron transfer 12

Supplementary Figure 5. Fast kinetic analysis of electron transfer rates in wt Na^+-NQR and Na^+-NQR variant NqrD-C29A lacking the $[2Fe\text{-}2S]_{NqrD\text{-}E}$ cluster 13

Cryo-EM map sharpening and density modifications 14

Supplementary Figure 6. Illustration of map improvements by density modification and map sharpening procedures 15

Cross-linking mass spectrometry confirm conformational changes of Na^+-NQR in solution 16

Supplementary Table 4. Calculated electron transfer rates between redox cofactors of Na^+-NQR..17

References 18

Movement of ferredoxin-like domain of NqrF

In the different cryo-EM and X-ray structures reported here, the ferredoxin-like domain of NqrF resides in different positions (Supplementary Fig. 1) illustrating that this domain is rather flexibly linked between the transmembrane helix and the FNR-like domain. E.g., aligning the NqrF subunit on the transmembrane helix, the Cα positions of the ferredoxin-like domain in the X-ray structure versus the cryo-EM structure with NADH and Q2 differ by a distance of 4-8 Å Angstrom. The position of the [2Fe-2S] cluster differs in these structures by 4.5 Å (Supplementary Fig. 1). Thus, the ferredoxin-like domain is flexibly tethered between the FAD-binding domain and the transmembrane helix allowing for large translational freedom, whereas the FNR-like domain interacts with NqrA that serves as a pivot point for rotational movements. This arrangement is key for domain motion of NqrF upon binding of NADH. The cryo-EM structure of Na^+-NQR reacted with NADH at 2.55 Å resolution shows that the ferredoxin-like domain of NqrF harbouring a [2Fe-2S] cluster approaches the membrane in a tilting motion, while the FNR-like domain of NqrF slides sideways to make space for the ferredoxin-like domain (Supplementary Video 1, Supplementary Fig. 1).

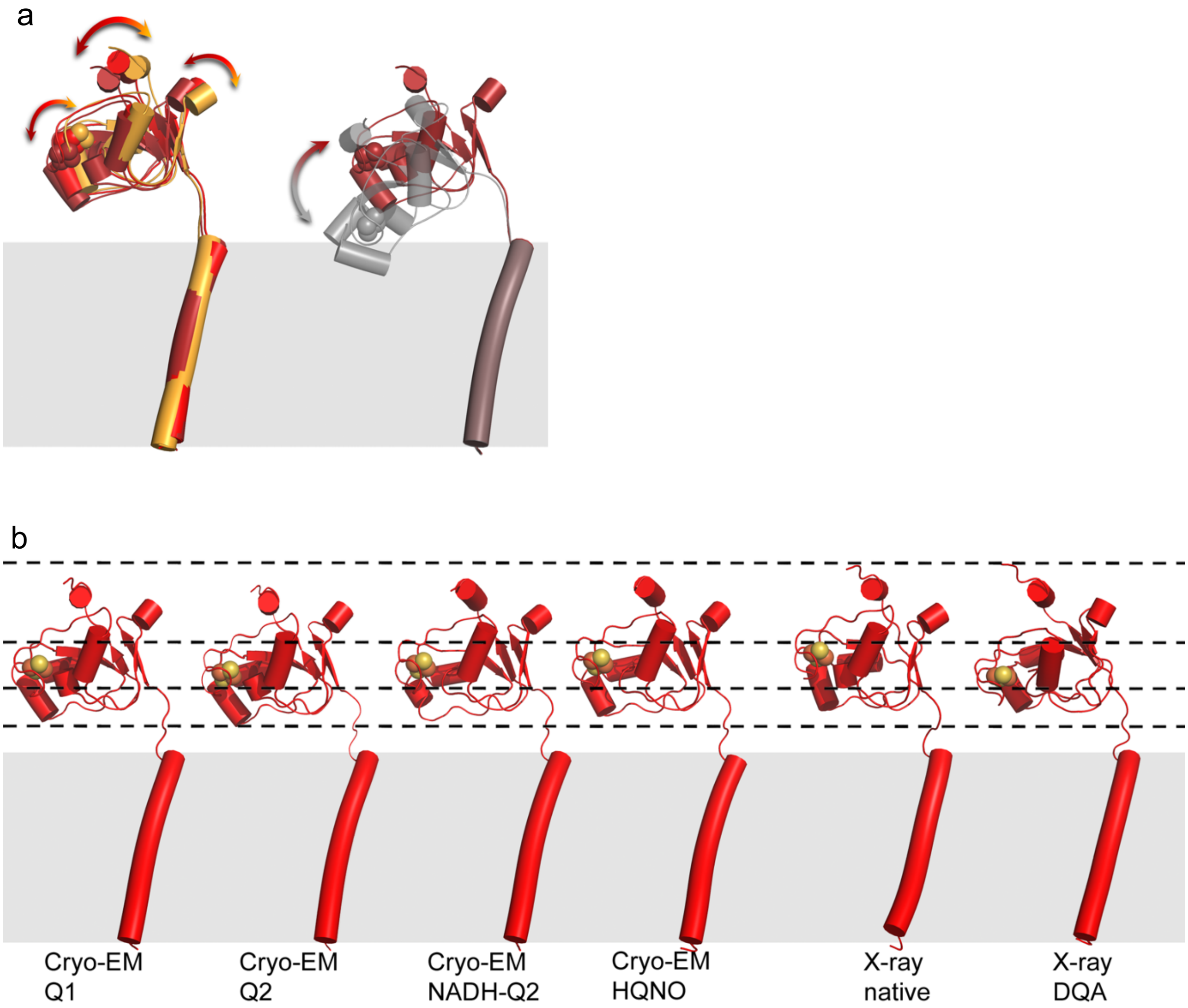

Supplementary Figure 1. Structural snapshots of rotational flexibility of the ferredoxin-like domain of NqrF. The ferredoxin-like domain of NqrF resides at different positions in the different cryo-EM and X-ray structures. **a,** Left: Structural alignment of the NqrF ferredoxin domain and the transmembrane helix domain. The FAD containing FNR-like domain is omitted for clarity. There is a rotational and a transversal movement of the ferredoxin domain relative to the transmembrane helix. For clarity, only the X-ray structure (orange), the cryo-EM structure with inhibitor HQNO (red) and the X-ray structure with inhibitor DQA (dark red) are shown. Right: extrapolation (grey) of the movement of the ferredoxin-like domain towards the membrane plane. **b,** Different conformations of the NqrF ferredoxin domain observed in this study with respect to the transmembrane helix. Broken lines indicate most distant and most proximate position of ferredoxin domain from the membrane, and most distant and proximate position of the [2Fe-2S] cluster from the membrane.

Analysis of the intramembranous [2Fe-2S] cluster in NqrD-E

In order to study the [2Fe-2S] clusters in NqrF or in NqrD-E individually, we have generated variants that lack either the [2Fe-2S] cluster in NqrF (NqrF-C70A) or the [2Fe-2S] cluster in NqrD-E (NqrD-C29A). These variants show only half of the Fe or acid labile sulphide content compared to wildtype Na^+-NQR (Supplementary Table 1) and largely reduced quinone reduction activity (Supplementary Table 2). Wildtype and variants of Na^+-NQR were analysed by a complementary set of several spectroscopic techniques including circular dichroism (CD), EPR, ^{57}Fe Mössbauer, and Fe Kα high-energy fluorescence detected (HERFD) X-ray absorption spectroscopy (XAS).

Supplementary Table 1. Detection of iron and acid-labile sulphide in wt Na^+-NQR and variants

The amount of iron and acid-labile sulphide was determined in Na^+-NQR and its variants. The amount of Fe found with inductively coupled plasma resonance mass spectroscopy was correlated to the sulphur content in the same sample. Acid-labile sulphide was determined colorimetrically. Mean values from three technical replicates of three independent protein preparations (n = 9) and standard deviations are given. APS reductase from *Desulfovibrio desulfuricans* served and bovine serum albumin served as controls.

**NQR variants**	**Mol Fe : mol protein**	**Mol S^{2-} : mol protein**	**Mol S^{2-} : mol Fe**
Wildtype	3.1 ± 0.5	3.6 ± 0.3	1.04
NqrF-C70A	1.4 ± 0.1	2.1 ± 0.1	0.95
NqrD-C29A	1.6 ± 0.2	2.4 ± 0.1	0.90
NqrE-C120S	1.4 ± 0.2	2.3 ± 0.1	0.83
NqrD-C29A/NqrE-C120A	1.3 ± 0.1	2.3 ± 0.3	0.78
**Control proteins**			
APS-reductase	n.d.	7.3 ± 0.1	n.d.
Bovine serum albumine	n.d.	0.63 ± 0.2	n.d.

Supplementary Table 2. Electron transfer activity of wt Na^+ -NQR and variants

Sodium dependent oxidation of NADH and reduction of ubiquinone-1 by Na^+-NQR was determined spectrophotometrically at 340 nm or 282 nm, respectively. The reaction was started with the addition of Na^+-NQR in the presence of saturating concentrations of ubiquinone-1 (UQ-1) (0.1 mM) and NADH (0.15 mM). The residual Na^+ concentration in the assay buffer was 20 μM Na^+. Standard derivations and mean of n= independent 3 experiments are given.

	Specific activity (μmol mg^{-1} min^{-1})			
	No NaCl added		30 mM NaCl	
	NADH	UQ-1	NADH	UQ-1
Wildtype	47.2 ± 3.1	6.5 ± 0.6	63.9 ± 3.1	13.2 ± 0.7
NqrF-C70A	3.4 ± 0.1	0.7 ± 0.1	4.0 ± 0.2	1.1 ± 0.1
NqrD-C29A	15.2 ± 0.1	2.2 ± 0.3	22.2 ± 0.8	3.3 ± 0.4
NqrE-C120S	25.7 ± 0.6	3.4 ± 0.1	33.2 ± 0.2	3.7 ± 0.2
NqrD-C29A/ NqrE-C120A	25.2 ± 0.7	3.3 ± 0.1	33.5 ± 1.4	3.6 ± 0.3

HERFD-XAS Fe K-edge and analysis of the [2Fe-2S] clusters in NqrF and NqrD-E

The Fe K- pre-edge XAS at 7112 eV of the NqrD-C70A sample was slightly shifted to higher energy indicating a distortion of the geometry of the $[2Fe\text{-}2S]_{NqrD\text{-}E}$ cluster (Supplementary Fig. 2a). Further evidence for such a distorted geometry comes also from the low field of X-band EPR spectra (see below and Supplementary Data Fig. 2 b-d). The EXAFS of both NqrF-C70A and NqrD-C29A exhibit an intense scattering shell in the non-phase shift corrected Fourier transform of the EXAFS at a radial distance of 1.90 Å that corresponds to degenerate Fe-S scattering interactions, (Supplementary Fig. 2a). This interaction is generally well-fit with a four-fold degenerate Fe-S scattering path at mean scattering distance of 2.28 Å (Fits 1 and 5, Supplementary Table 3, Supplementary Fig. 3). Furthermore, a second less intense scattering shell is observed at a longer radial distance of 2.40 Å that corresponds to an Fe-Fe scattering interaction. Inclusion of a single Fe-Fe scattering interaction at 2.7 Å significantly improves the EXAFS fits of both NqrF-C70A and NqrD-C29A (Fits 2 and 6, respectively), as evidenced by a significant reduction of the χ^2 value. These EXAFS fits clearly assign the iron cofactors of NqrF-C70A and NqrD-C29A as [2Fe-2S] clusters.

EPR spectroscopic analysis of the [2Fe-2S] clusters in NqrF and NqrD-E

The EPR spectra of the dithionite reduced [2Fe-2S] cluster in NqrF exhibits features resembling vertebrate-type ferredoxins and has been described by us in previous studies[1,2] (Supplementary Fig. 2b). EPR spectra of the so far uncharacterized intramembranous cluster in NqrD-E exhibited a weak signal with two distinct features (Supplementary Fig. 2b,e). The prominent feature at $g \sim 2.01$ is consistent with a microwave power saturated radical signal, while the feature at $g \sim 1.94$ was only observed under relatively high microwave powers, indicative for a fast-relaxing species. The assigned *g*-values of the NqrD-E cluster are similar to that observed for the NqrF cluster[23], but we do note the potentially very different relaxation behaviours of the two clusters as indicated by differences in microwave power required for observation. The high-field feature (Supplementary Fig. 2c,d) resembles the $g_{\perp}$ of various [2Fe-2S] clusters[21,22], however, a distinct corresponding $g_{||}$ feature of an axial EPR spectrum in the expected range of 2.01 to 2.06 is not observed. At low field an additional feature at g=6.4 was observed indicating the presence of some cluster in a higher spin state than S=1/2 (Supplementary Fig. 2 c,d). The feature is reminiscent of a [2Fe-2S] cluster with an non-Cys ligand in the coordination sphere that exhibits a S=9/2 giving rise to a EPR transition with a feature at g=6.8[3].

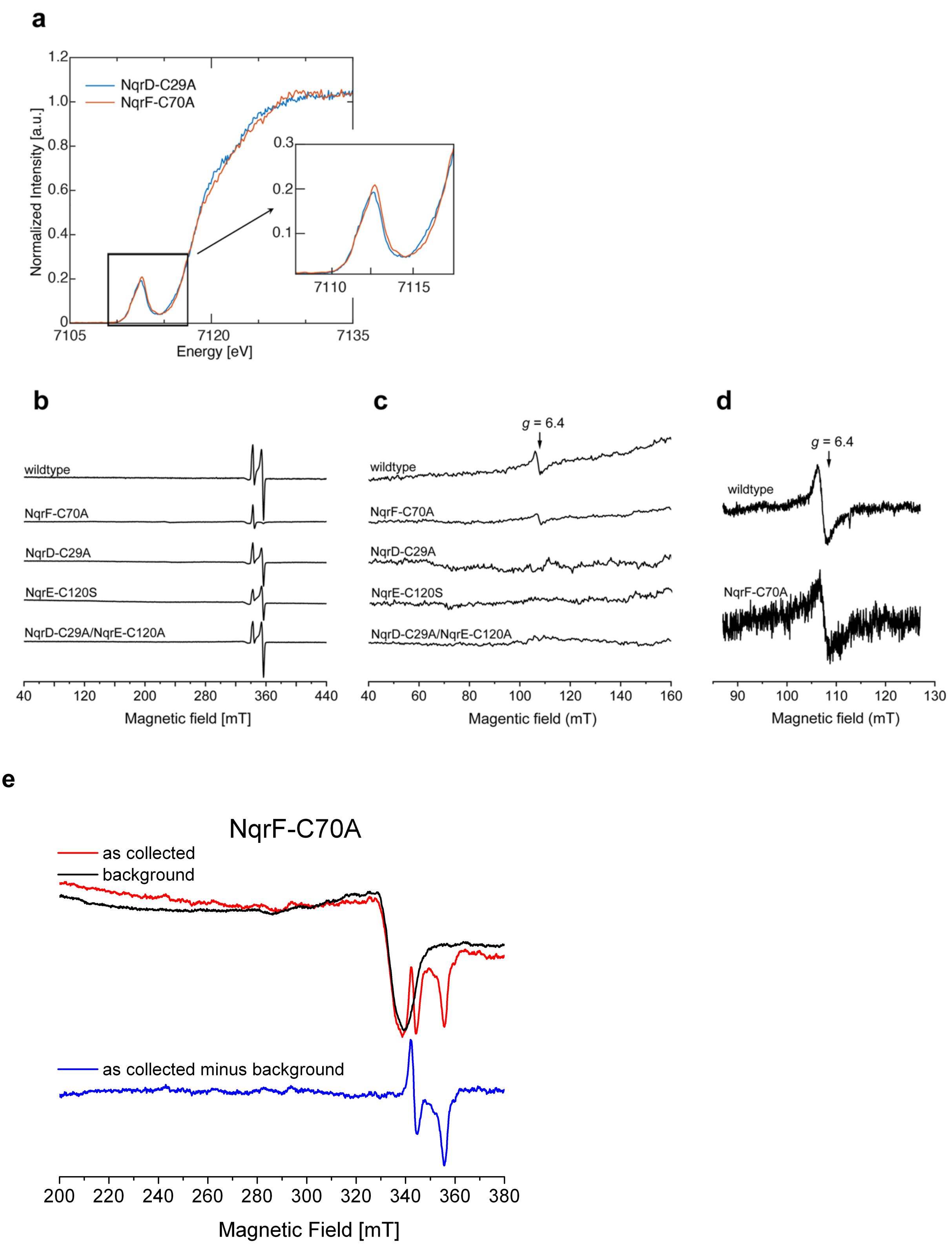

Supplementary Figure 2. HERFD-XAS Fe K-edge and EPR spectra of Na^+-NQR wildtype and Na^+-NQR variants (Figure legend details see below)

Supplementary Figure 2. HERFD-XAS Fe K-edge and EPR spectra of Na^+-NQR wildtype and Na^+-NQR variants (above)

a, HERFD-XAS Fe K-edge of Na^+-NQR variants NqrD-C29A and NqrF-C70A, either lacking the cluster NqrD-E or the cluster in NqrF, respectively. **b-d,** X-band EPR spectra of dithionite reduced Na^+-NQR wildtype and variants lacking either the $[2Fe\text{-}2S]_{NqrF}$ or the $[2Fe\text{-}2S]_{NqrD\text{-}E}$ cluster. **b**, EPR spectra recorded at 0.2 mW microwave power exibit a radical signal in all samples. In variants lacking $[2Fe\text{-}2S]_{NqrD\text{-}E}$ cluster (NqrD-C29A, NqrE-C120S, NqrD-C29A/NqrE-C120A) the $[2Fe\text{-}2S]_{NqrF}$ cluster is clearly visible, whereas no signal is visible at low microwave power in the variant lacking $[2Fe\text{-}2S]_{NqrF}$ cluster (NqrF-C70A). No signal is visible in the low field region. **c-d**, Low field region of the samples at high microwave power (10-25 mW). Na^+-NQR wildtype and Na^+-NQR variant lacking $[2Fe\text{-}2S]_{NqrF}$ cluster (NqrF-C70A) exhibit a weak signal at g=6.4. **e**, Raw, as collected EPR spectrum of NqrF-C70A and a background EPR spectrum of a water blank sample, both collected under the same conditions: 10 K; 9.644 GHz; 10 mW microwave power; 7.46 G modulation amplitude; 100 kHz modulation frequency; 81.92 ms conversion time; 20.48 ms time constant. The subtraction of the two spectra is offset vertically for clarity. EPR settings b-d: microwave frequency=9.64 GHz, temperature=10 K, time constant=0.02 s, modulation amplitude=0.75 mT, modulation frequency=100MHz. All spectra in b recorded at microwave power=0.2 mW. Spectra in **c** were recorded with 20 mW for wildtype and variant NqrF-C70A, and with 25 mW for variants NqrD-C29A, NqrE-C120S, NqrD-C29A/NqrE-C120A. Spectra in d were recorded at microwave power of 10 mW.

Supplementary Table 3. EXAFS Fit Parameters.

NqrF-C70A								
Fit #	Path	***N***	*R* (Å)	± (Å)	σ^2 (Å^2)	± (Å^2)	ΔE_0[a)]	χ^2
1	Fe-S	4	2.275	0.011	0.0057	0.0007	4.032	30.46
2	Fe-S	4	2.279	0.007	0.0057	0.0005	4.967	12.70
	Fe-Fe	1	2.729	0.014	0.0053	0.0014		
**NqrD-C29A**								
Fit #	Path	***N***	*R* (Å)	± (Å)	σ^2 (Å^2)	± (Å^2)	ΔE_0[a)]	χ^2
3	Fe-S	4	2.280	0.011	0.0064	0.0007	4.265	32.40
4	Fe-S	4	2.284	0.007	0.0064	0.0005	5.164	13.65
	Fe-Fe	1	2.740	0.014	0.0056	0.0014		

a) ΔE_0 value is reported as shift from set E_0 = 7118.55 eV; Same ΔE_0 is used for all paths in a single fit.

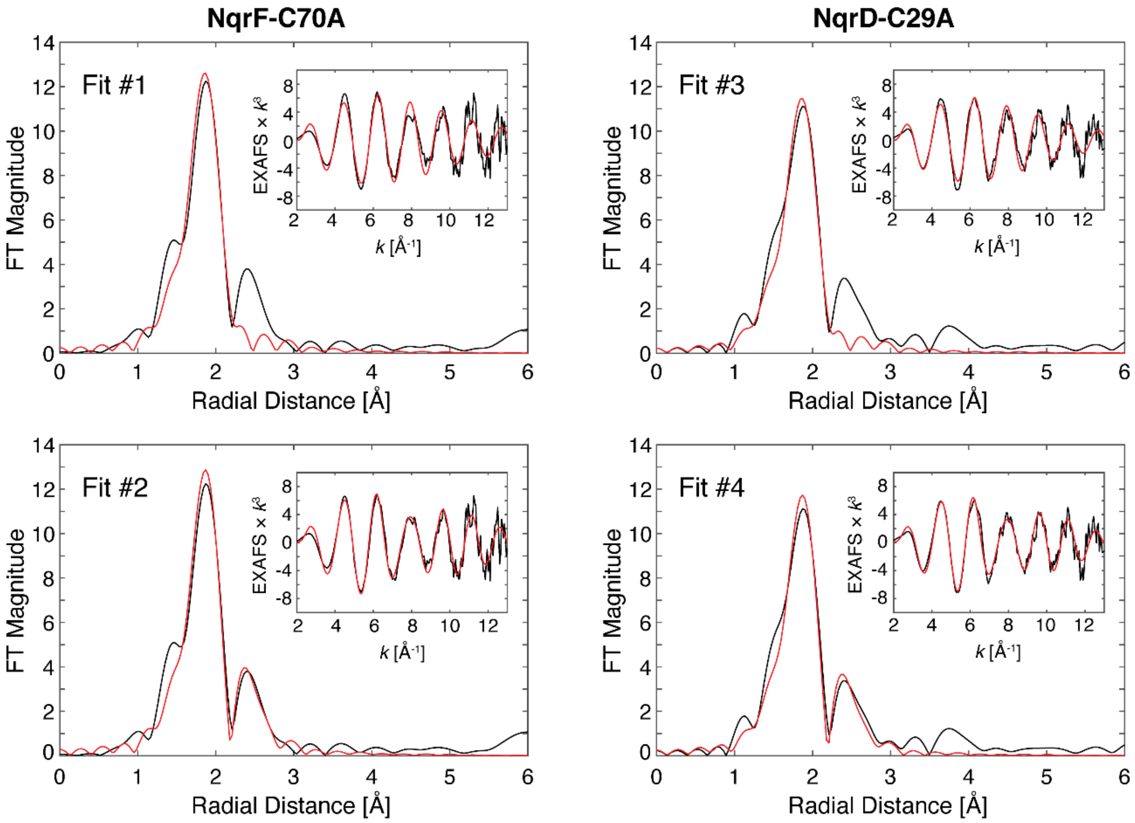

Supplementary Figure 3. EXAFS of NqrF-C70A and NqrD-C29A.

The non-phase shifted Fourier transforms of the k^3-weighted EXAFS (insets) is presented (black) with respective fits (red). Fit parameters are outlined in Supplemental Data Table 3. All Fourier transforms were taken over a k-range of 2-13 Å^{-1}.

UV-visible absorption spectroscopy of wt-NQR and of a Na^+-NQR variant

Comparison of UV-visible spectra of wt-NQR and of a Na^+-NQR variant lacking the $[2Fe\text{-}2S]_{NqrD\text{-}E}$ cluster (Cys29 in NqrD exchanged to Ala; NqrD-C29A) showed significant differences. The calculated difference spectrum (wt minus variant) exhibits features typical for a [2Fe-2S] cluster with absorption maxima at 335 nm and 473 nm and a shoulder at 575 nm (Supplementary Fig. 5a). In particular, the absorption at 575 nm is specific for the intramembranous $[2Fe\text{-}2S]_{NqrD\text{-}E}$ cluster and gives also rise to a band in the CD spectrum (Supplementary Fig. 4b). This absorption band is remote of the flavin absorption features and therefore well-suited to follow changes in the redox state of the intramembranous $[2Fe\text{-}2S]_{NqrD\text{-}E}$ cluster.

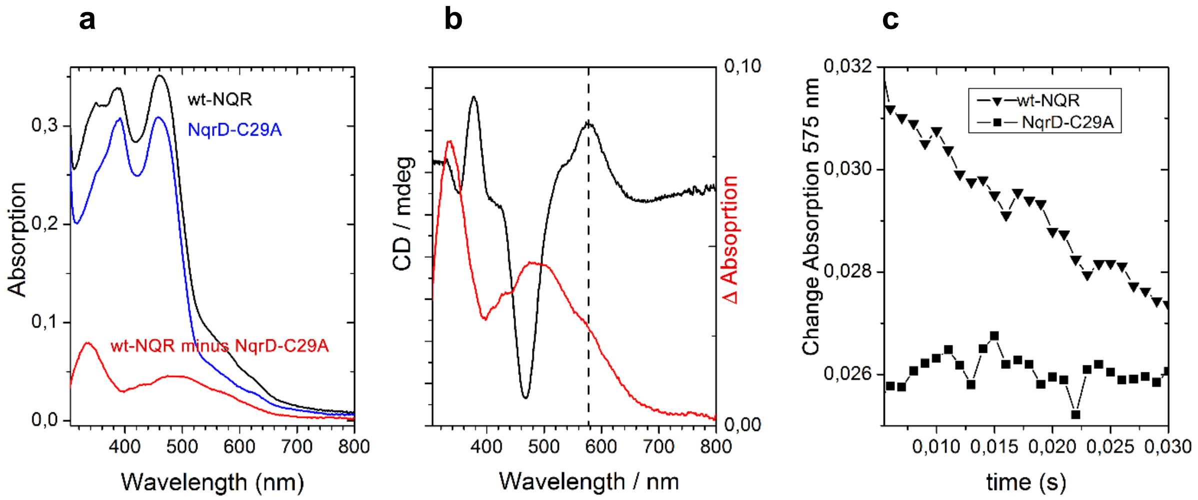

Supplementary Figure 4. Spectroscopic and fast kinetic analysis of wt Na^+-NQR and Na^+-NQR variants reveal specific features of the $[2Fe\text{-}2S]_{NqrD\text{-}E}$

a, UV-visible spectra of wt-NQR (black) and variant NqrD-C29A (blue) lacking the $[2Fe\text{-}2S]_{NqrD\text{-}E}$ cluster. The difference spectrum (red) shows features typical of a [2Fe-2S] cluster. **b,** Overlay of difference spectrum from A and CD spectrum of a variant (NqrF-C70A) containing only $[2Fe\text{-}2S]_{NqrD\text{-}E}$ cluster. The position of a shoulder at 575 nm in the UV-vis spectrum and of a band in the CD is indicated by a broken line. This feature is specific to $[2Fe\text{-}2S]_{NqrD\text{-}E}$ in Na^+-NQR. **c,** Change in absorption at 575 nm in wt Na^+-NQR (triangles) and NqrD-C29A (squares) upon reduction of the protein complex by NADH.

The $[2Fe\text{-}2S]_{NqrD\text{-}E}$ centre is conducting transmembrane electron transfer

The position of the novel $[2Fe\text{-}2S]_{NqrD\text{-}E}$ cluster implies that it is crucial for transmembrane electron transfer. Electrons are transferred from cytoplasmic $[2Fe\text{-}2S]_{NqrF}$ cluster of NqrF via the intramembranous $[2Fe\text{-}2S]_{NqrD\text{-}E}$ to the periplasmic FMN of NqrC.

$$[2Fe\text{-}2S]_{NqrF} \rightarrow \mathbf{[2Fe\text{-}2S]_{NqrD\text{-}E}} \rightarrow FMN_{Periplasm\ NqrC} \rightarrow FMN_{Periplasm\ NqrB}$$

In order to proof transmembrane electron transfer via $[2Fe\text{-}2S]_{NqrD\text{-}E}$, we studied electron transfer in Na^{+}-NQR by stopped-flow kinetic measurements. To trace such changes during electron transfer we applied stopped-flow fast kinetic measurements (Supplementary Fig. 4c and Supplementary Data Fig. 5).

Reaction of wt-NQR with NADH at 20° C results in rapid reduction of the FAD in NqrF subunit already within the dead time of the instrument. Subsequently, electrons are transferred from FAD to the other cofactors (Supplementary Fig. 5a). The first electron steps can be described by first order kinetics and were fitted by linear regression in semi-logarithmic plots. Reduction of the [2Fe-2S] cluster in NqrF within 5 milliseconds was observed. Clearly, also the intramembranous $[2Fe\text{-}2S]_{NqrD\text{-}E}$ cluster was reduced (Supplementary Fig. 5a) during course of the reaction, which was confirmed by a decrease of the absorption at 575 nm (Supplementary Fig. 5c). A third rate observed represents the reduction of the FMN in NqrC.

In contrast the reaction of variant NqrD-C29A with NADH resulted only in partial reduction of the cofactors (Supplementary Fig. 5b). No further spectral change was observed even after 1 second. The variant lacking [2Fe-2S] in NqrD-E showed like wt Na^{+}-NQR rapid reduction of FAD and subsequent electron transfer to the [2Fe-2S] located in NqrF. However, electron transfer in NqrD-C29A lacking $[2Fe\text{-}2S]_{NqrD\text{-}E}$ was interrupted at NqrF and no further reduction of FMN in NqrC was observed. No change in absorption was absorbed at 575 nm, in agreement with the absence of cluster $[2Fe\text{-}2S]_{NqrD\text{-}E}$ (Supplementary Fig. 4c).

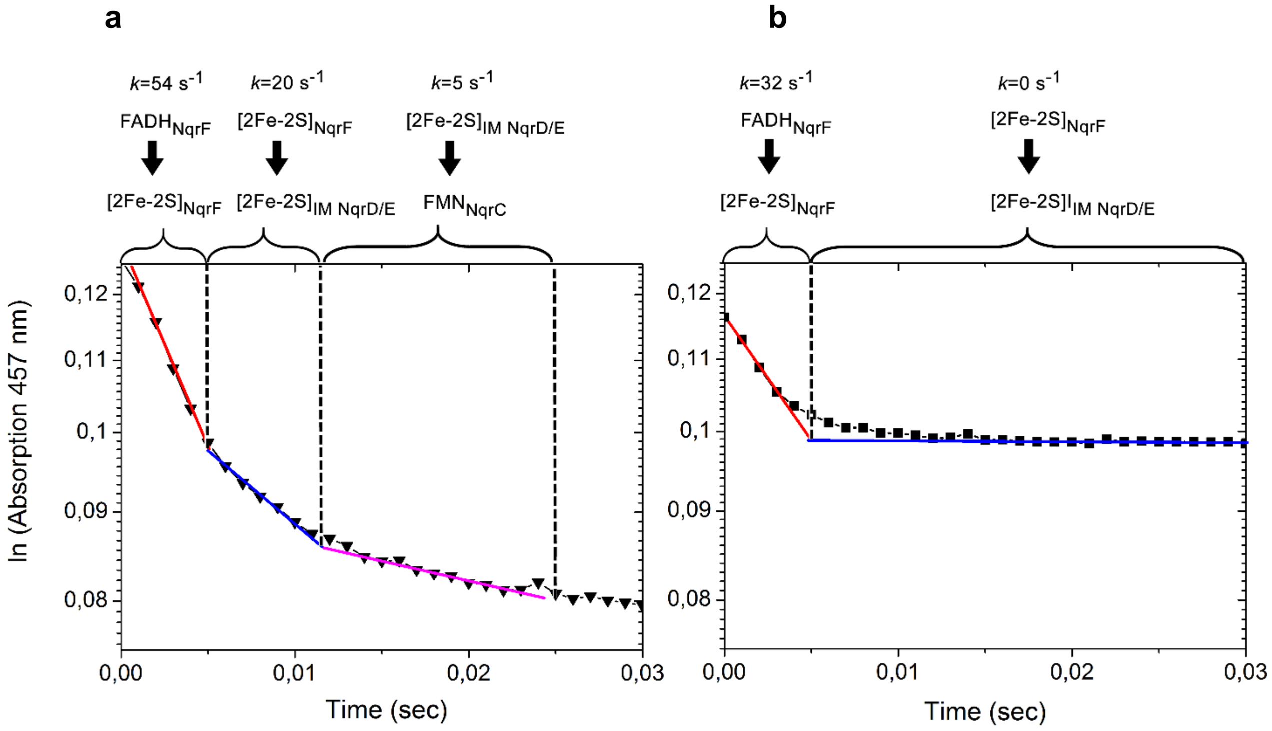

Supplementary Figure 5. Fast kinetic analysis of electron transfer rates in wt Na^{+}-NQR and Na^{+}-NQR variant NqrD-C29A lacking the [2Fe-2S]$_{NqrD-E}$ cluster

a, Fast kinetic time course of absorption upon reduction with NADH in wt Na^{+}-NQR. In a semi-logarithmic plot of ΔA457 nm versus time one can fit at least three different 1st order reaction rates corresponding to (i) the reduction of the [2Fe-2S] in NqrF, (ii) subsequent reduction of intramembranous [2Fe-2S] in NqrD-E and (iii) reduction of the FMN in NqrC. **b,** Fast kinetic time course of absorption upon reduction with NADH in variant NqrD-C29A. Only the electron transfer from FAD to the [2Fe-2S] in NqrF is observed.

Cryo-EM map sharpening and density modifications

Different map sharpening procedures were applied throughout the entire process of model building and the outcome of the different sharpening procedures have been extensively compared to yield the best possible maps. Best results with respect to recognisable details were obtained by density modifications and *phenix.resolve_cryo-em* [4] and resampling of the final map at 0.3 - 0.5 Å/pixel using *relion_image_handler* [5] (Supplementary Fig. 7 a-c). We have applied regularly *phenix.auto_sharpen* [6], *LocalDeBlur* [7], *LocScale* [8] or *DeepEMhancer* [9]. *phenix.auto_sharpen* gave often satisfying results, while e.g. *DeepEMhancer* yielded maps with better connectivity, in particular for map regions which have been weak due to flexibility of the domains, like e.g. NqrF (Supplementary Fig. 6i-m).

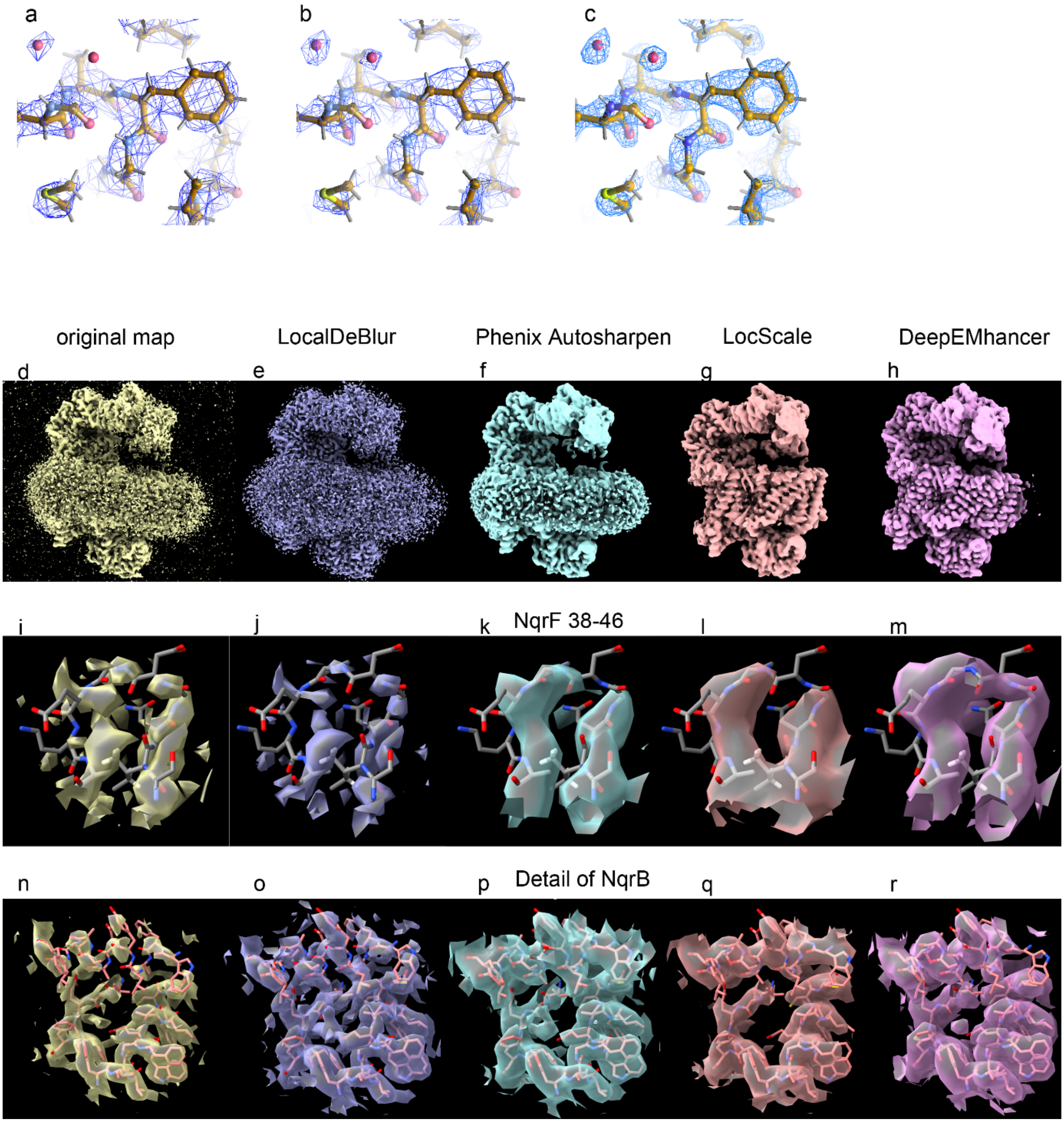

Supplementary Figure 6. Illustration of map improvements by density modification and map sharpening procedures.

a, Original map of Na^{+}-NQR with UQ-1 at 2.56 Å resolution around Phe238 in NqrB. **b**, After density modification with *phenix.resolve_cryo_em* the nominal resolution is 2.1 Å. **c**, After resampling of the map, features like backbone carbonyls, additional water molecules or the aromatic nature of the benzyl of phenylalanine become clearly visible. Further map modifications and sharpening procedures resulted in better interpretable maps for regions of low resolution or weak density. **d-h**, View of the map around entire Na^{+}-NQR after different map modifications. **i-m**, The different procedures yielded maps with better connectivity, like for the flexibly linked ferredoxin-domain of subunit NqrF. A loop comprising residues NqrF 38-46 of the ferredoxin-like domain is shown. **n-r**, With respect to map clarity and visibility of sidechains, map sharpening with *phenix.auto_sharpen* returned well-interpretable maps as shown here for a region of NqrB.

Cross-linking mass spectrometry confirm conformational changes of Na^+-NQR in solution

Cross-linking mass spectrometry (XL-MS) experiments were performed on two different states of the complex, in absence and in presence of the inhibitor DQA (see Methods). The amine-reactive reagent, disuccinimidyl suberate (DSS), was used to covalently link spatially proximal residues within and between different subunits, and cross-linking sites were identified by liquid chromatography-tandem mass spectrometry. An overview of the identified cross-links is provided in Supplementary Data Set 1. In addition, we compared the abundances of cross-linked peptide pairs in the two states (±DQA) by label-free quantification using *xTract* to identify regions of potential conformational changes upon inhibitor binding.

Among the quantitative differences observed (Supplementary Data Set 2), changes in three particular regions are noteworthy: First, within NqrA, many cross-links increased or decreased in abundance in presence of DQA. In absence of major conformational changes within this subunit, as judged from cryo-EM data, this suggests that residues within that subunit become more or less accessible to DSS upon DQA binding, likely due to domain movements within NqrF relative to NqrA. Second, an inter-subunit cross-link between NqrE and NqrF increases in the +DQA state, which reflects a more favourable orientation (likely closer proximity of the reactive sites) of the NqrF domain towards NqrE. Third, within NqrC, many cross-links are less abundant (and none increase in abundance) in the inhibitor-bound state. This likely reflects rearrangements within NqrC that correspond to an "opening" of the subunit, therefore increasing the distances between cross-linked residues.

Two independent cross-links between NqrF and NqrD and NqrF and NqrE, respectively, confirm that the ferredoxin-like domain of NqrF approaches NqrD-E at a distance that can be bridged by the cross-linker (distance < 30 Å Cα-Cα). These data confirm that the ferredoxin-like domain of NqrF approaches NqrD-E at a distance of approximately 14 Å corresponding to the length of the linker. This movement relocates the [2Fe-2S] cluster of the ferredoxin-like domain into electron transfer distance to the FeS centre in NqrD-E.

Supplementary Table 4. Calculated electron transfer rates between redox cofactors of Na^+-NQR

	Cry-EM structure Na^+-NQR with NADH and UQ-2		X-ray structure Na^+-NQR	
redox pair	distance / Å *1	calculated electron transfer rate / s^{-1} *2	distance / Å *1	calculated electron transfer rate / s^{-1} *2
FAD_{NqrF} – $[2Fe\text{-}2S]_{NqrF}$	9.4	$7.37 \cdot 10^{6}$	10.8	$1.07 \cdot 10^{6}$
$[2Fe\text{-}2S]_{NqrF}$ – $[2Fe\text{-}2S]_{NqrD\text{-}E}$	32.2	$5.50 \cdot 10^{-8}$	34.1	$3.98 \cdot 10^{-8}$
$[2Fe\text{-}2S]_{NqrD\text{-}E}$ – FMN_{NqrC}	27.3	$1.35 \cdot 10^{-5}$	7.2	$1.55 \cdot 10^{7}$
FMN_{NqrC} – FMN_{NqrB}	5.7	$1.81 \cdot 10^{8}$	22.1	$2.61 \cdot 10^{-2}$
FMN_{NqrB} – $riboflavin_{NqrB}$	8.2	$1.63 \cdot 10^{6}$	7.6	$3.73 \cdot 10^{6}$
$riboflavin_{NqrB}$ – UQ-2 $_{NqrB}$	11.5	$8.07 \cdot 10^{4}$	n.d.	n.d.

*[1] shortest edge-to-edge distance between non-hydrogen atoms of redox cofactors

*[2] calculated according to [10,11] $\log k_{et} = 13 - 0.6\,(R - 3.6) - 3.1 \cdot (\Delta G - \lambda)^2/\lambda$

k_{et} = electron transfer rate (s^{-1}), R = edge-to-edge distance (Å), ΔG = difference free energy (eV),
λ = reorganization energy (eV)

References

1. Türk, K. *et al.* NADH oxidation by the Na^+-translocating NADH:quinone oxidoreductase from *Vibrio cholerae*: functional role of the NqrF subunit. *J. Biol. Chem.* **279,** 21349–21355 (2004).
2. Lin, P.-C. *et al.* A vertebrate-type ferredoxin domain in the Na^+-translocating NADH dehydrogenase from *Vibrio cholerae*. *J. Biol. Chem.* **280,** 22560–22563 (2005).
3. Subramanian, S. *et al.* Spectroscopic and redox studies of valence-delocalized $Fe_2S_2^{(+)}$ centers in thioredoxin-like ferredoxins. *J. Am. Chem. Soc.* **137,** 4567–4580 (2015).
4. Terwilliger, T. C., Sobolev, O. V., Afonine, P. V., Adams, P. D. & Read, R. J. Density modification of cryo-EM maps. *Acta Crystallogr. D Biol. Crystallogr.* **76,** 912–925 (2020).
5. Kimanius, D., Dong, L., Sharov, G., Nakane, T. & Scheres, S. H. W. New tools for automated cryo-EM single-particle analysis in RELION-4.0. *Biochem. J.* **478,** 4169–4185 (2021).
6. Liebschner, D. *et al.* Macromolecular structure determination using X-rays, neutrons and electrons: recent developments in Phenix. *Acta Crystallogr. D Biol. Crystallogr.* **75,** 861–877 (2019).
7. Ramírez-Aportela, E. *et al.* Automatic local resolution-based sharpening of cryo-EM maps. *Bioinformatics (Oxford, England)* **36,** 765–772 (2020).
8. Jakobi, A. J., Wilmanns, M. & Sachse, C. Model-based local density sharpening of cryo-EM maps. *eLife* **6** (2017).
9. Sanchez-Garcia, R. *et al.* DeepEMhancer: a deep learning solution for cryo-EM volume post-processing. *Communications biology* **4,** 874 (2021).
10. Moser, C.C. and Dutton P.L. Engineering protein structure for electron transfer function in photosynthetic reaction centers. *Biochimica et Biophysica Acta* **1101**, 171–17 (1992).
11. Page, C.C., Moser, C.C., Chen, X. & Dutton, P.L. Natural engineering principles of electron tunnelling in biological oxidation-reduction. Nature **402**, 47–52 (1999)
