# Supplementary material for: Conformational coupling of redox-driven Na+-translocation in Vibrio cholerae NADH:quinone oxidoreductase
Source: Nat Struct Mol Biol. 2023 Sep 14;30(11):1686–94. doi: 10.1038/s41594-023-01099-0 (PMC10643135; doi:10.1038/s41594-023-01099-0)
Supplement: Supplementary file 2 — Reporting Summary [file 41594_2023_1099_MOESM2_ESM.pdf]

nature portfolio

Corresponding author(s): Günter Fritz, Janet Vonck, Julia Fritz-Steuber

Last updated by author(s): May 2, 2023

Reporting Summary

Nature Portfolio wishes to improve the reproducibility of the work that we publish. This form provides structure for consistency and transparency in reporting. For further information on Nature Portfolio policies, see our Editorial Policies and the Editorial Policy Checklist.

Statistics

For all statistical analyses, confirm that the following items are present in the figure legend, table legend, main text, or Methods section.

n/a	Confirmed	
☐	☒	The exact sample size (*n*) for each experimental group/condition, given as a discrete number and unit of measurement
☐	☒	A statement on whether measurements were taken from distinct samples or whether the same sample was measured repeatedly
☐	☒	The statistical test(s) used AND whether they are one- or two-sided<br>*Only common tests should be described solely by name; describe more complex techniques in the Methods section.*
☒	☐	A description of all covariates tested
☒	☐	A description of any assumptions or corrections, such as tests of normality and adjustment for multiple comparisons
☐	☒	A full description of the statistical parameters including central tendency (e.g. means) or other basic estimates (e.g. regression coefficient) AND variation (e.g. standard deviation) or associated estimates of uncertainty (e.g. confidence intervals)
☐	☒	For null hypothesis testing, the test statistic (e.g. *F*, *t*, *r*) with confidence intervals, effect sizes, degrees of freedom and *P* value noted<br>*Give P values as exact values whenever suitable.*
☒	☐	For Bayesian analysis, information on the choice of priors and Markov chain Monte Carlo settings
☒	☐	For hierarchical and complex designs, identification of the appropriate level for tests and full reporting of outcomes
☒	☐	Estimates of effect sizes (e.g. Cohen's *d*, Pearson's *r*), indicating how they were calculated

Our web collection on statistics for biologists contains articles on many of the points above.

Software and code

Policy information about availability of computer code

Data collection	Data were collected at synchrotron sources: 1) PETRA-3 beamline P14 using MXCUBE 2) at Swiss-Light -Source beamlines X06SA and X06DA using DA+ for data acquisition. Cryo-EM images were recorded using EPU (version 2.1).
Data analysis	All X-ray images were processed with XDS (version Jan 10, 2022 BUILT=20220820) and scaled with XSCALE (version Jan 10, 2022 BUILT=20220820) . For analysis of anomalous scattering the program anode was used. Molecular replacement was performed with Phaser (version 2.8.3). X-ray structures were refined using Refmac5 (version 5.8.350) and phenix.refine (version 1.20.1) and Coot (version 0.9.8.1) was used for manual rebuilding. Anomalous difference maps were calculated with the program anode (version 2013/1).<br>Cryo-EM images were processed using RELION4. Particles were picked using Topaz (version 0.2.4).Contrast transfer function was estimated using CTFFIND4.1 inside RELION4. Cryo-EM maps were sharpened with LocScale and phenix.resolve_cryo_em (version 1.20.1). Partial models were generated using Alphafold2. The models were refined using phenix.real_space_refine (version 1.20.1) and Coot (version 0.9.8.1) for manual rebuilding. Restraints used during refinement for ubiquinone-1, ubiquinone-2, HQNO, and riboflavin were generated with acedrg (version 246) from smiles codes; for energy minimization Refmac5 (version 5.8.0350) was used. Restraints for the covalent link between FMN and threonine were generated using acedrg. The cif file was edited manually using angles and distances obtained after energy minimization with gamess (version 18 AUG 2016 (R1)). Harmonic restraints used during refinement in phenix.refine (version 1.20.1) and phenix.real_pace_refine7 (version 1.20.1) for the coordination and geometry of the [2Fe 2S] clusters and coordination of the Na+ ions were generated by phenix.elbow (version 1.20.1) Putative ions in the structures were analyzed with WASP (version 1.0). Putative ion channels were identified with HOLE (version 2.2.005). Docking calculations were performed with PLANTS (version 1.2), SMINA (version Oct 15 2019, based on AutoDock Vina 1.1.2) and VINAXB (based on AutoDock Vina 1.1.2) . Cross-linked peptides from mass spec analysis were identified using xQuest (version 2.1.5) and quantification of cross-links was performed using xTract (version 1.0.2). KM values were fitted using Origin 2019.

For manuscripts utilizing custom algorithms or software that are central to the research but not yet described in published literature, software must be made available to editors and reviewers. We strongly encourage code deposition in a community repository (e.g. GitHub). See the Nature Portfolio guidelines for submitting code & software for further information.

Data

Policy information about availability of data

All manuscripts must include a data availability statement. This statement should provide the following information, where applicable:

- Accession codes, unique identifiers, or web links for publicly available datasets
- A description of any restrictions on data availability
- For clinical datasets or third party data, please ensure that the statement adheres to our policy

Cryo-EM density maps have been deposited in the Electron Microscopy Data Bank under accession numbers EMD-15088 (Na+-NQR native), EMD-15091 (Na+-NQR with ubiquinone-1), EMD-15090 (Na+-NQR with ubiquinone-2), EMD-15089 (Na+-NQR with ubiquinone-2 and NADH), EMD-15092 (Na+-NQR with DQA), EMD-15093 (Na+-NQR with HQNO). Cryo EM model coordinates have been deposited in the Protein Data Bank under accession numbers 8A1T (Na+-NQR native), 8A1W (Na+-NQR with ubiquinone-1), 8A1V (Na+-NQR with ubiquinone-2), 8A1U (Na+-NQR with ubiquinone-2 and NADH), 8A1X (Na+-NQR with DQA), 8A1Y (Na+-NQR with HQNO).
X-ray structure coordinates and structure factors for the entire complex of Na+-NQR and of individual subunit NqrF (residues 129–408) and NqrF-F406A variant (residues 129–408) with and without substrate NADH have been deposited in the Protein Data Bank. The PDB accession codes are 8ACY (Na+-NQR updated entry), 8ACW (Na+-NQR new entry), 8ACY (Na+-NQR with DQA), 8AD4 (subunit NqrF with NADH), 8AD3 (subunit NqrF-F406A), 8AD5 (subunit NqrF-F406A with NADH).
The cross-linking mass spectrometry data have been deposited to the ProteomeXchange Consortium via the PRIDE48 partner repository with the dataset identifier PXD039289.
The structure of Na+-NQR (pdb code 4P6V) was used as an initial model for model building of cryo-EM and X-ray structures. For molecular replacement the structure of NqrF FAD domain (pdb code 4U9U) was used as search model. Models were retrieved from the pdb database (https://www.rcsb.org/)

Research involving human participants, their data, or biological material

Policy information about studies with human participants or human data. See also policy information about sex, gender (identity/presentation), and sexual orientation and race, ethnicity and racism.

Reporting on sex and gender	does not apply
Reporting on race, ethnicity, or other socially relevant groupings	does not apply
Population characteristics	does not apply
Recruitment	does not apply
Ethics oversight	does not apply

Note that full information on the approval of the study protocol must also be provided in the manuscript.

Field-specific reporting

Please select the one below that is the best fit for your research. If you are not sure, read the appropriate sections before making your selection.

☒ Life sciences ☐ Behavioural & social sciences ☐ Ecological, evolutionary & environmental sciences

For a reference copy of the document with all sections, see nature.com/documents/nr-reporting-summary-flat.pdf

Life sciences study design

All studies must disclose on these points even when the disclosure is negative.

Sample size	Sample size is defined by the samples themselves. Crystals for X-ray analysis contain billions of molecules. Cryo-EM single particle requires several hundred thousands molecules.
Data exclusions	No data were excluded. In X-ray data integration severe outliers of spots intensity are identified by data processing software and excluded. These are usually only in the range of tens to few hundreds diffraction spots compared to millions of spots integrated per dataset.
Replication	Experimental procedures are highly reproducible. X-ray structure analysis involved measurement of several hundred crystals with highly similar properties. Cryo-EM analysis was performed with several grids, each containing several hundreds to thousands holes.
Randomization	Randomization was not relevant for this study. Samples are highly homogeneous.

Blinding	Blinding was not relevant for this study. Samples are highly homogeneous.

Reporting for specific materials, systems and methods

We require information from authors about some types of materials, experimental systems and methods used in many studies. Here, indicate whether each material, system or method listed is relevant to your study. If you are not sure if a list item applies to your research, read the appropriate section before selecting a response.

Materials & experimental systems

n/a	Involved in the study
☒	☐ Antibodies
☒	☐ Eukaryotic cell lines
☒	☐ Palaeontology and archaeology
☒	☐ Animals and other organisms
☒	☐ Clinical data
☒	☐ Dual use research of concern
☒	☐ Plants

Methods

n/a	Involved in the study
☒	☐ ChIP-seq
☒	☐ Flow cytometry
☒	☐ MRI-based neuroimaging
