# Supplementary figures and images for: Conformational coupling of redox-driven Na+-translocation in Vibrio cholerae NADH:quinone oxidoreductase
Source: Nat Struct Mol Biol. 2023 Sep 14;30(11):1686–94. doi: 10.1038/s41594-023-01099-0 (PMC10643135; doi:10.1038/s41594-023-01099-0)

Coomassie stained protein bands on SDS-PAGE

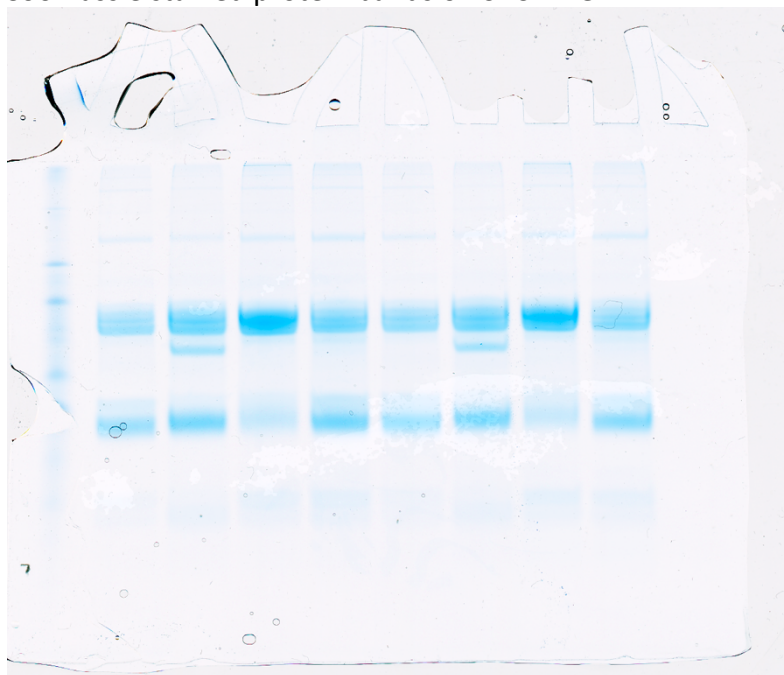

Flavin fluorescence of protein bands on SDS-PAGE

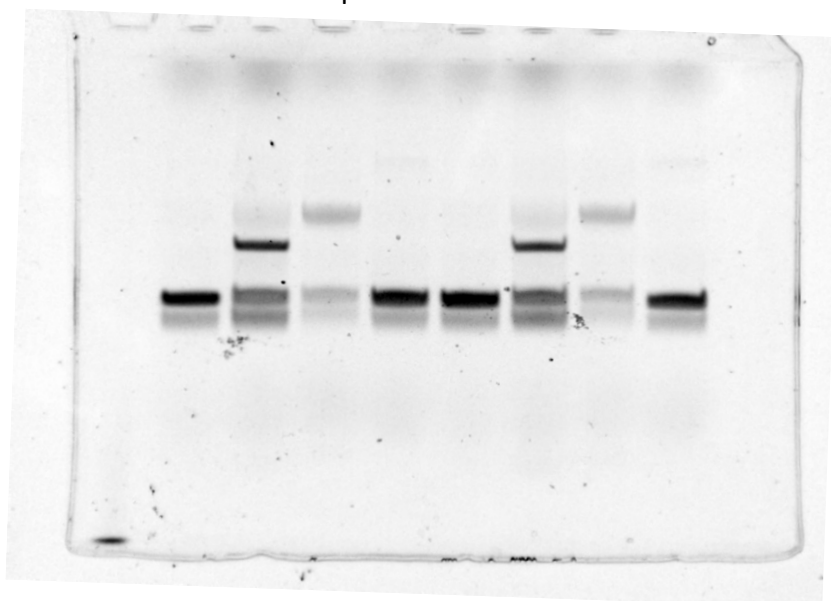

Supplement: Supplementary file 11 — Unprocessed Coomassie-stained gel and flavin fluorescence of gel. [file 41594_2023_1099_MOESM11_ESM.pdf]
